# AI-driven credibility profiling of real-world patient experiences suggests overlooked kidney stone therapies warrant further investigation

**DOI:** 10.21203/rs.3.rs-9388837/v1

**Published:** 2026-04-17

**Authors:** Alfredo Parra-Hinojosa, Andrés Gómez-Emilsson, Daniel C. Elton

**Affiliations:** 1 Qualia Research Institute, Sacramento, CA, USA; 2 National Human Genome Research Institute, National Institutes of Health, Bethesda, MD, USA

**Keywords:** Kidney Calculi, Nephrolithiasis, Patient Outcome Assessment, Prophylaxis, Cost-Benefit Analysis, Kidney stones, urolithiasis, Phyllanhus Niruri, nephrology

## Abstract

Thousands of patients share their testimonials of kidney stone treatment efficacy online routinely, often with the hope of helping other patients understand what may help alleviate the severe suffering inflicted by the condition. Such testimonials generate vast data with potential hypothesis-generating insights that remain largely untapped. For this study, 4,899 publicly available online testimonials of nine different kidney stone products were collected across three different platforms: WebMD, Amazon, and Reddit. Using a state-of-the-art Large Language Model, these subjective reports were classified along multiple dimensions, focusing on reported effectiveness and rate of adverse effects. A methodology to minimize biases and extract statistically significant data from online testimonials is presented. Odds ratios and p-values were calculated using *Phyllanthus niruri* as the baseline. *Phyllanthus niruri* demonstrated significantly higher reported effectiveness across all platforms (27.6%–87.4%) compared to most other treatments, including allopurinol, tamsulosin, hydrochlorothiazide, and potassium citrate. On Amazon, Rowatinex showed the highest effectiveness rate (90.0%). Both *Phyllanthus niruri* and Rowatinex exhibited remarkably low adverse effect profiles across all platforms (3.9% and 4.5% weighted average, respectively), with Rowatinex notably reporting no severe adverse effects. The consistency of these findings across different platforms suggests that patient-reported outcomes for *Phyllanthus niruri* and Rowatinex may warrant further clinical investigation. The rising incidence and debilitating nature of kidney stones underscore the need for research into additional prophylactic and metaphylactic treatments beyond lifestyle interventions.

## Introduction

Kidney stones (KS) can lead to the most painful experiences people endure. A majority of patients consider KS to be the worst pain they have ever experienced, including women who have experienced the pain of childbirth.[ Additionally, patients report strong feelings of stress and anxiety about recurrence [2] and lower health-related quality of life [3] While the Global Burden of Disease data indicates declining age-standardized rates worldwide [4] several regions and countries (including the United States) show concerning increases in prevalence over recent decades [5] The absolute burden continues to rise, posing major wellbeing and healthcare challenges [4]

The American Urological Association acknowledges that, relative to its burden, research into KS disease prevention remains “surprisingly sparse”.[6] At the same time, official preventative strategies tend to focus on minimizing stone *recurrence* [6,7], but since first-time stone formers have much higher recurrence risk, [8] more primary prevention strategies are warranted. However, current primary prevention strategies tend to emphasize lifestyle approaches such as increased fluid intake, dietary modifications, and weight management.[9] Pessimistically, insights from behavioral economics indicate that lifestyle changes are difficult to adopt and adhere to, which may partly explain the lack of success in significantly reducing the global burden of KS.[10] Too many people only start taking preventative steps once it is too late.[11]

Online KS support groups have proliferated on social media in recent years, hosting an overwhelming abundance of patient reports. Recent advances in artificial intelligence (in particular, Large Language Models or LLMs) now make it possible to make sense of such data with a fraction of resources, revealing insightful patterns and preferences. In this study, real-world data (RWD) in the form of testimonials and product reviews from three online platforms (WebMD, Amazon, and Reddit) were collected and classified to understand which KS treatments are reported as most effective by patients. This approach aligns with the FDA's Real-World Evidence Program framework, which acknowledges the value of evidence derived from sources other than traditional clinical trials to generate testable hypotheses for randomized controlled trials.[12] Recent emphasis on patient engagement in KS research validates the complementary importance of patient-reported outcomes.[13]

This study argues that online testimonials and product reviews can be used as a source for medical hypothesis generation. While online testimonials can be susceptible to various biases, *a priori*, highly efficacious treatments with few adverse effects should be reviewed more positively than mediocre treatments with significant adverse events. A rigorous methodology is presented that accounts for biases to a large degree.

## Materials and methods

### Addressing limitations of real-world data from online platforms

The use of RWD from online testimonials is subject to several inherent limitations, which this study aimed to control for. Selection biases (wherein patients with extreme positive or negative outcomes are more likely to share reviews), placebo effect biases, and astroturfing concerns (posting inauthentic reviews to create a false impression of popular support) were controlled by comparing multiple treatments *within* the same platform and *across* three different platforms, allowing for the assessment of relative, rather than absolute, outcomes. Furthermore, a potential "pro-supplement" or “anti-pharma” bias on WebMD was controlled by analyzing reviews of two widely-used supplements (melatonin and ashwagandha) to serve as controls. An additional stratified statistical analysis was performed on reviews deemed to be of particularly high quality, such as when reviewers gave extensive background information and detailed protocols and outcomes.

This methodological approach is particularly valuable for substances like *P. niruri*, where the path to large-scale RCTs may be hindered by a lack of patentability and subsequent commercial funding. This study aims to provide preliminary evidence to help bridge this epistemological gap and justify further, more rigorous investigation.

### Data collection

Online testimonials of nine treatments for KS were collected and classified, both standard (namely, hydrochlorothiazide, potassium citrate, allopurinol, and tamsulosin) and alternative (namely, *Phyllanthus niruri* aka chanca piedra, Rowatinex^®^, Phosfood^®^, black seed, and *Garcinia cambogia* extract—a source of hydroxycitric acid). Publicly available testimonials were collected from three different platforms: WebMD^®^, Amazon^®^, and Reddit^®^.

WebMD is a health news and database platform where, among others, users can rate and review drugs, vitamins, and supplements (not tied to specific brands). Reviews of five KS products were extracted using WebMD’s interface to filter for condition = KS, hydrochlorothiazide (n=20), potassium citrate (n=24), allopurinol (n=13), tamsulosin (n=22), and *P. niruri* (n=87). Additionally, reviews of black seed (n=77) and garcinia (n=892) were collected to analyze their adverse event profiles (none of the reviews were specifically for KS). Finally, to further investigate whether WebMD reviews might exhibit potential “pro-supplement” or “anti-pharma” biases, reviews of two widely-used non-KS supplements were analyzed: ashwagandha (n=271) and melatonin (n=161).

On Amazon, users can rate and review specific branded products. Four KS products were included in the analysis: potassium citrate (eight different brands, n=133), *P. niruri* (nine different brands, n=1,193), Rowatinex (single proprietary brand, n=90), and Phosfood (single proprietary brand, n=40). For potassium citrate and *P. niruri*, the most popular brands (with the largest number of reviews) were selected.

Finally, Reddit is a discussion platform organized by thematic sub-communities where users can, for instance, share experiences and seek advice from other KS patients. All posts and comments in the community r/KidneyStones mentioning any of the nine KS products (including alternative names and common misspellings) were included: hydrochlorothiazide (n=46), potassium citrate (n=509), allopurinol (n=49), tamsulosin (n=1,134), *P. niruri* (n=492), Rowatinex (n=21), Phosfood (n=2; removed from analysis), black seed (n=17), and garcinia (n=38).

All data were collected using Microsoft Playwright 1.49.1.

### Data processing & classification

A state-of-the-art LLM (Anthropic’s Claude 3.5 Sonnet) was instructed in October 2024 to classify all testimonials across dozens of dimensions, such as: pain reduction, breaking/shrinking/softening/dissolution of stones, quicker stone expulsion, patient history of recurrence, overall review quality, adverse events mentioned, dose used, stone characteristics, etc. For most dimensions, the LLM returned coded values (e.g., for “pain reduction”, it returned “1” if the reviewer reported pain improvements, “0” if pain worsened, and “null” if no claim was made). A randomly chosen subset of 112 reviews was first classified manually to verify the accuracy of LLM classification. A 93% agreement was observed along the most important dimension (reported effectiveness), which was considered sufficient for this work. Finally, all reported adverse events were categorized (again via LLM) into three levels of severity (mild, moderate, and severe).

Two Amazon brands of *P. niruri* had hundreds of 5-star reviews, but only a maximum of approximately 100 of them are publicly accessible. This resulted in low star ratings being overrepresented. Weighting corrections were implemented to account for this sampling bias, by comparing scraped counts of star ratings to the actual distribution displayed on Amazon.

### Outcome measures

Two outcome measures were of primary interest: (1) the proportion of reviews mentioning a product helping overall with KS and (2) the proportion of reviews mentioning adverse events caused by that product. These metrics were reported separately for each of the three platforms. Additionally, both metrics are reported with an additional stratification by review quality (namely, all reviews vs. high-quality reviews only). High-quality reviews were identified using LLM based on the degree of specificity, detail, and coherence criteria of each review.

Additionally, odds ratios (ORs) with 95% CIs were calculated for each comparator versus *P. niruri* on the same platform.

### Statistical analysis

All analyses were conducted using *Python* (3.12.8) with *pandas* (2.2.3) for data manipulation, *numpy* (2.0.1) for numerical operations, *scipy* (1.15.2) for statistical tests, *statsmodels* (0.14.4) for regression models and confidence intervals, and *plotly* (5.24.1) for visualization. The entire analysis pipeline was implemented in *Jupyter Notebook* to ensure reproducibility.

Primary metrics included 95% confidence intervals (CIs) using Wilson’s score method.

To quantify the relative effectiveness and adverse events profile of different KS treatments, comparative analyses were conducted using *P. niruri* as the reference treatment across all platforms. *P. niruri* was selected based on its prominence in alternative medicine discussions and consistent presence across all three platforms, which allows for standardized comparisons.

For each platform, ORs were calculated by comparing the likelihood of reported effectiveness and adverse events between *P. niruri* and each KS treatment. Comparisons were structured as 2x2 contingency tables (treatment type × outcome), using a hierarchical method selection algorithm to determine the most appropriate statistical test for each comparison:

- If both events and non-events per arm exceeded 10 observations, logistic regression with weights was used.
- If the expected cell frequencies (calculated using scipy’s *expected_freq*) were below 5, Fisher's exact test was used.
- If any cell had fewer than 5 observations, only descriptive statistics were reported, without inferential testing.

For comparisons meeting the sample size threshold for regression, weighted logistic regression models were used with robust standard errors (HC0) via *statsmodel*. The model included treatment type as the sole independent variable (with *P. niruri* as the reference category) and either effectiveness or adverse events as the binary dependent variable. Odds ratios were extracted by exponentiating the regression coefficients, with 95% confidence intervals similarly derived from the coefficient confidence intervals. P-values were obtained from Wald tests on the regression coefficients.

Fisher's exact tests were conducted using scipy.stats’ *fisher_exact* function, which provides odds ratios, confidence intervals, and two-sided p-values.

Statistical significance is indicated by asterisks (* p<0.05, ** p<0.01, *** p<0.001). P-values were not adjusted for multiple comparisons, as this analysis was considered hypothesis-generating rather than confirmatory.

## Results

Remarkably, on WebMD, 87.4% of reviews of *P. niruri* mentioned it being effective in treating KS, significantly higher than allopurinol (38.5%, p<10^−3^), tamsulosin (27.3%, p<10^−3^), hydrochlorothiazide (40.0%, p<10^−3^), and potassium citrate (45.8%, p<10^−3^) (Table 1a). Additionally, only 6.9% of *P. niruri* reviews mentioned adverse events, significantly lower than allopurinol (53.8%, p<10^−3^), black seed (26.0%, p<10^−2^), garcinia (48.5%, p<10^−3^), hydrochlorothiazide (65.0%, p<10^−3^), and potassium citrate (62.5%, p<10^−3^) (Table 1b). Insufficient high-quality reviews prevented statistical comparison of effectiveness or adverse events, though descriptive statistics favored *P. niruri*.

On Amazon, 71.0% of reviews of *P. niruri* mentioned it being effective in treating KS, similar to Phosfood (72.5%) and potassium citrate (66.9%) but lower than Rowatinex (90.0%, p<10^−3^). All four products had a similarly low rate of reported adverse events (2.7% for *P. niruri* compared to 2.2% for Rowatinex, 2.3% for potassium citrate, and 7.5% for Phosfood), but ORs and p-values were not calculated for any pair due to the low number of observed events (including for high-quality reviews).

On Reddit, posts mentioning *P. niruri* were significantly more likely to report effectiveness in treating KS (27.6%) than allopurinol (14.3%, p<.05), tamsulosin (11.2%, p<10^−3^), hydrochlorothiazide (13.0%, p<.05), and potassium citrate (13.4%, p<10^−3^). *P. niruri* was also more effective than black seed (11.8%) and Rowatinex (23.8%), but not significantly. Only garcinia (31.6%) was reported as more effective than *P. niruri*, but not significantly. High-quality reviews, where available, support these trends. The rate of adverse events reported for *P. niruri* (6.5%) was lower than all products except black seed (0%), reaching significance when compared to tamsulosin (21.9%, p<10^−3^) and potassium citrate (13.4%, p<10^−3^). Significance was not reached when comparing high-quality reviews only (and the trend reversed slightly in favor of allopurinol, potassium citrate, and Rowatinex).

The overall lower rates on Reddit compared to WebMD and Amazon are due to Reddit's discussion format, where posts often consist of questions or comments rather than reviews. For instance, of the 17 mentions of “black seed,” only two consisted of actual testimonials.

Figures 1a,b visually summarize the primary metrics for each of the three platforms.

**Fig. 2:**
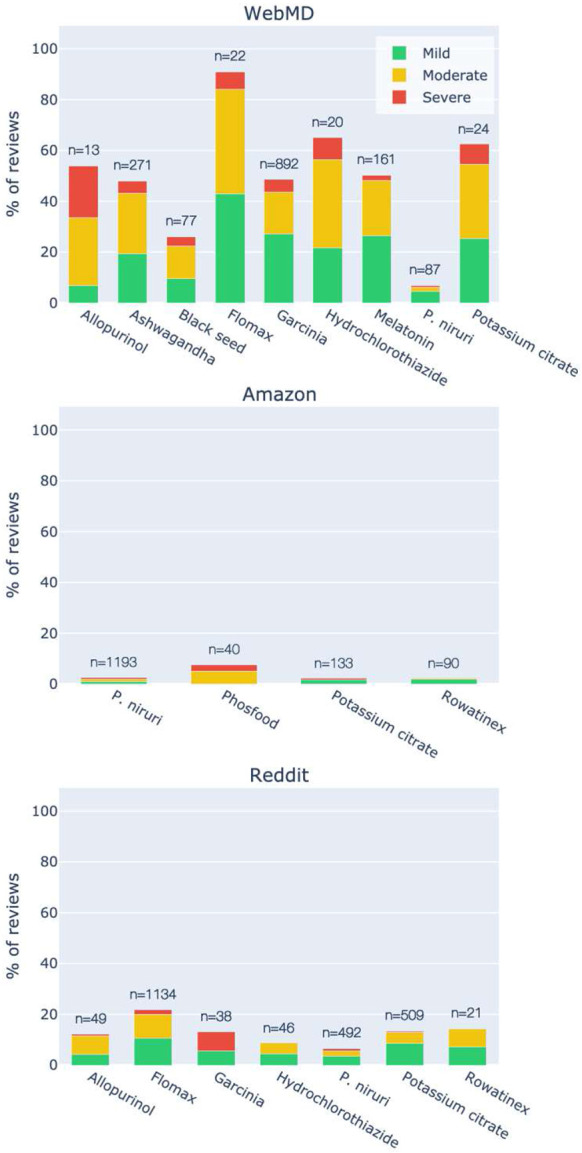
The fraction of reviews that reported adverse events, classified by severity.

The classification of reported adverse events by severity (mild, moderate, severe) is summarized in Figure 2. *P. niruri* reviews had the lowest weighted average of reported adverse events across platforms (3.9%), followed by Rowatinex (4.5%). Rowatinex also stood out as the only product for which no severe adverse events were reported, followed by potassium citrate (0.70%) and *P. niruri* (0.74%). The large fraction of adverse events reported for ashwagandha and melatonin compared to *P. niruri* (48.0% and 50.3% respectively, p<10^−3^) speaks against potential “anti-pharma” or “pro-supplement” biases on WebMD.

## Discussion

Online testimonials show KS patients favor *P. niruri* and Rowatinex over other standard and alternative treatments, based on the reported effectiveness and low rate of adverse events. Neither of these two are considered first-line treatments for KS. Their exclusion from treatment guidelines likely reflects insufficient evidence (given the small number of publications on both) rather than proven ineffectiveness or safety concerns.

A 2020 meta-analysis of *P. niruri* [14] found only two RCTs [15,16] and one prospective controlled study [17] of *P. niruri*’s effects on humans. Two of those three [16,17] reported effects on stone size and number, with pooled data including 89 patients treated with *P. niruri* and 92 controls who received placebo. The pooled data revealed significant decreases in standardized mean stone size (−0.39 cm, 95% CI: −0.68, −0.09, p=0.01) and stone number (−0.38, 95% CI: −0.68, −0.09, p=0.01). The third study reported a statistically significant increase in stone-free rates (for lower caliceal stones) from treatment with *P. niruri* followed by extracorporeal shock wave lithotripsy (ESWL) (93.7% vs 70.8% in the control group, p=0.01). [15] Studies of *P. niruri* have consistently reported a lack of serious adverse events, even at very high doses, consistent with our findings and attesting to *P. niruri’*s favorable safety profile.[18]

Evidence of the efficacy of Rowatinex is similarly sparse, documented primarily in Romics et al.’s 2010 placebo-controlled RCT.[19] In their study of 222 patients with a 12 -week active phase and 2-week follow-up, they found that significantly more patients were stone-free after treatment with Rowatinex following ESWL: 67.9% in the intent-to-treat group compared to 50.0% in the placebo group (p=0.0009) and 78.4% in the per-protocol group compared to 52.2% in the placebo group (p=0.0004). A significant decrease in median time to stone-free status was also observed in both groups compared to placebo. The authors noted that Rowatinex was well tolerated and safe, and concluded that their findings corroborated preclinical studies attesting to the treatment’s antilithogenic, antibacterial, anti-inflammatory, spasmolytic, and analgesic effects.

While the mechanisms of action of *P. niruri* and Rowatinex are still not fully understood, available evidence suggests that they may be particularly efficacious at preventing stone formation, [19,20] although likely through separate mechanisms given their distinct chemical composition (*P. niruri* being phytochemically rich, particularly in polyphenols, and Rowatinex being essentially a blend of terpenes).

It has been hypothesized that the constituents of *P. niruri* can prevent stone formation by inhibiting the binding of calcium to oxalate to form calcium oxalate, which has been observed in vitro [21] and in a rat model.[22]

The potential efficacy of *P. niruri* and Rowatinex may be explained by surface area-to-volume principles in crystal dissolution kinetics. Fragmented stones present greater surface area for biochemical interaction compared to intact stones of equivalent mass, potentially enhancing the ability of active compounds to disrupt crystal lattice structures and inhibit further aggregation, a hypothesis consistent with in vitro findings that *P. niruri* significantly increases the rate of crystal nucleation while decreasing the rate of crystal growth.[23] This suggests that prophylactic and metaphylactic use of such antilithogenic agents may be particularly effective at interrupting early-stage nucleation events and preventing pathological processes such as Randall's plaque formation and Randall's plug development [24] Periodic prophylactic administration could theoretically reset the lithogenic potential of the urinary environment. Staying stone-free may prove significantly easier than becoming stone-free after a first KS event.

KS treatments can often act synergistically. For example, the European Association of Urology states that combining tamsulosin with chemolysis for uric acid stones >8mm in particular is more effective than each treatment alone. A 2024 study by Di Mauro et al. [25] showed that a combination of tamsulosin with three herbal extracts (boldine, *P. niruri*, and *Ononis spinosa*) was significantly more effective than tamsulosin alone in treating kidney stones. Additional longitudinal studies combining different treatments may be warranted, for instance with oxalate-degrading microorganisms [26] or with *Terminalia arjuna* plant extracts. [27]

Some have suggested discouraging patients from taking *P. niruri* on the basis of limited published evidence, [28] a recommendation we question since the intense suffering associated with KS should factor prominently in risk-benefit assessments of treatments with encouraging safety profiles and patient-reported effectiveness. As an illustrative example, even in one case where a reviewer on WebMD reported a severe adverse event possibly attributed to *P. niruri*, the patient considered the trade-off worthwhile:

Been using Chanca Piedra for over a decade - works great preventing Kidney Stones or getting rid of them quickly when they pop up. However, […] I have definitely been diagnosed with Meniere's Disease […]. My ENT and my Therapist pretty much diagnosed the dizziness to loose ear crystals. Ear crystals are chemically similar to kidney stones, so I suspect the Chanca Piedra may be related to that. Even so, I would rather have recurring bouts of dizziness than kidney stones.

It is clear from the many reviews analyzed that KS patients will try almost anything to find relief from the excruciating pain, described by another online reviewer as follows:

Can vouch, the pain is so extreme it is almost like a living thing; a living thing that does not want to kill you, sadly. Oh, no, death is too good for you. This thing is all about the torture. […] That pain in your side is hard to describe to someone who has never had them. It is pure, unadulterated, undiffused, exquisite pain.

The consistent patient-reported benefits of *P. niruri* and Rowatinex suggest that these treatments may offer a low-risk add-on intervention with potential benefits. The much higher rates of adverse events reported for ashwagandha and melatonin, which are already very widely used by the general population and considered safe, offers some additional evidence (however minor) that the overly cautious stance on *P. niruri* or Rowatinex may be inconsistent with the widespread acceptance of other supplements that exhibit less favorable patient-reported safety profiles.

Lo et al. [29] highlight a paradox: despite significant progress in understanding stone pathophysiology, recommended preventive interventions have not changed for years, and recent evidence has even begun to challenge the efficacy of staple treatments such as thiazides. In the spirit of motivating future research directions, their review of 38 studies investigating non-guideline treatments for preventing calcium oxalate kidney stones found that tolvaptan, cranberry juice, magnesium citrate, ALLN-177, and malic acid had evidence to reduce urinary risk factors; SGLT2i, eicosapentaenoic acid, and ethane-1-hydroxy-1,1-disphosphonate (EHDP) had evidence to reduce stone formation; and *P. niruri*, rice bran, and magnesium hydroxide had evidence of both. Finally, other non-guideline treatments mentioned in the reviews analyzed in our study include marshmallow root, *Hydrangea* root, horsetail tea, and black radish.

## Conclusions

Real-world data in the form of online testimonials on WebMD, Amazon, and Reddit suggest KS patients find *P. niruri* and Rowatinex to be at least as effective as standard (and a few alternative) treatments, with few adverse events. The patient-reported benefits observed in this study, combined with limited but encouraging data from previous studies, strengthens the case for prioritizing these treatment options in future research efforts (including RCTs and mechanistic studies), thus expanding treatment options for KS patients, which may be necessary to make more rapid progress toward decreasing the burden of this debilitating condition.

LLM-driven data classification made it possible to analyze thousands of subjective reports with modest time investment and high accuracy. Beyond the specific findings of this study, this work illustrates the potential of AI-enabled approaches to extract insights from large volumes of unstructured data.

## Figures and Tables

**Fig. 1a:**
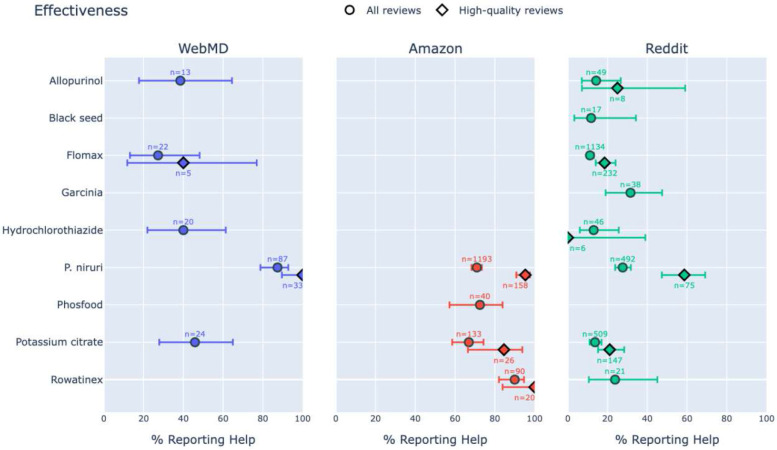
Effectiveness (proportion of reviews mentioning a product helping with KS) across platforms, with 95% CI, for products with ≥5 data points.

**Fig. 1b:**
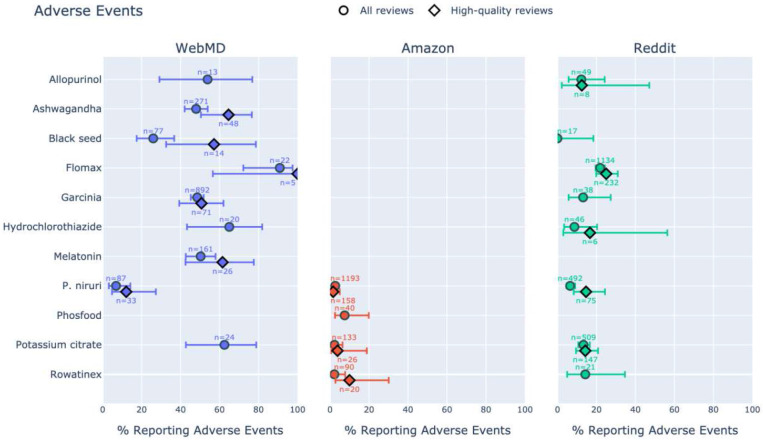
Adverse events (proportion of reviews mentioning any products causing adverse events) across platforms, with 95% CI, for products with ≥5 data points.

**Table 1:** Reported effectiveness of *P. niruri* (reference) vs other treatments (comparison). Data in square brackets refer to high-quality reviews only. OR > 1 means reviews of comparison treatment are more likely to report helping with KS than *P. niruri*

Platform	N_Reference_	Helped_Reference_	Comparison	N_Comparison_	Helped_Comparison_	Odds Ratio	Test
**WebMD**	87 [33]	87.4% (78.8-92.8) [100.0% (89.6-100.0)]	Allopurinol	13	38.5% (17.7-64.5)	0.09 (0.03–0.33) ***	Fisher
			Tamsulosin	22 [5]	27.3% (13.2-48.2) [40.0% (11.8-76.9)]	0.05 (0.02–0.17) *** [-]	Fisher [-]
			Hydrochlorothiazide	20	40.0% (21.9-61.3)	0.10 (0.03–0.29) ***	Fisher
			Potassium citrate	24	45.8% (27.9-64.9)	0.12 (0.04–0.34) ***	Logistic Regression
**Amazon**	1193 [158]	71.0% (68.3-73.5) [95.3% (90.8-97.7)]	Phosfood	40	72.5% (57.2-83.9)	1.08 (0.54–2.17)	Logistic Regression
			Potassium citrate	133 [26]	66.9% (58.5-74.3) [84.6% (66.5-93.8)]	0.83 (0.57–1.20) [-]	Logistic Regression [-]
			Rowatinex	90 [20]	90.0% (82.1-94.6) [100.0% (83.9-100.0)]	7.27 (3.62–14.61) *** [-]	Fisher [-]
**Reddit**	492 [75]	27.6% (23.9-31.8) [58.7% (47.4-69.1)]	Allopurinol	49 [8]	14.3% (7.1-26.7) [25.0% (7.1-59.1)]	0.44 (0.19–0.99) * [-]	Fisher [-]
			Black seed	17	11.8% (3.3-34.3)	-	-
			Tamsulosin	1134 [232]	11.2% (9.5-13.2) [18.5% (14.1-24.0)]	0.33 (0.25–0.43) *** [0.16 (0.09–0.28) ***]	Logistic Regression [Logistic Regression]
			Garcinia	38	31.6% (19.1-47.5)	1.21 (0.59–2.46)	Logistic Regression
			Hydrochlorothiazide	46 [6]	13.0% (6.1-25.7) [0.0%]	0.39 (0.16–0.95) * [-]	Fisher [-]
			Potassium citrate	509 [147]	13.8% (11.0-17.0) [21.1% (15.3-28.4)]	0.42 (0.30–0.58) *** [0.19 (0.10–0.35) ***]	Logistic Regression [Logistic Regression]
			Rowatinex	21	23.8% (10.6-45.1)	0.82 (0.29–2.28)	Fisher

**Table 2:** Reported adverse events caused by *P. niruri* (reference) vs other treatments (comparison). Data in square brackets refer to high-quality reviews only. OR > 1 means reviews in comparison are more likely to report adverse events than *P. niruri.*

Platform	N_Reference_	Adverse Events_Reference_	Comparison	N_Comparison_	Adverse Events_Comparison_	Odds Ratio	Test
**WebMD**	87 [33]	6.9% (3.2-14.2) [12.1% (4.8-27.3)]	Allopurinol	13	53.8% (29.1-76.8)	15.75 (4.00–61.98) ***	Fisher
			Ashwagandha	271 [48]	48.0% (42.1-53.9) [64.6% (50.4-76.6)]	12.45 (5.25–29.50) *** [-]	Fisher [-]
			Black seed	77 [14]	26.0% (17.5-36.7) [57.1% (32.6-78.6)]	4.74 (1.79–12.54) ** [-]	Fisher [-]
			Tamsulosin	22 [5]	90.9% (72.2-97.5) [100.0% (56.6-100.0)]	- [-]	- [-]
			Garcinia	892 [71]	48.5% (45.3-51.8) [50.7% (39.3-62.0)]	12.74 (5.50–29.49) *** [-]	Fisher [-]
			Hydrochlorothiazide	20	65.0% (43.3-81.9)	25.07 (7.27–86.44) ***	Fisher
			Melatonin	161 [26]	50.3% (42.7-57.9) [61.5% (42.5-77.6)]	13.67 (5.64–33.12) *** [-]	Fisher [-]
			Potassium citrate	24	62.5% (42.7-78.8)	22.50 (6.98–72.55) ***	Fisher
**Amazon**	1193 [158]	2.7% (1.9-3.7) [1.6% (0.5-5.0)]	Phosfood	40	7.5% (2.6-19.9)	-	-
			Potassium citrate	133 [26]	2.3% (0.8-6.4) [3.8% (0.7-18.9)]	- [-]	- [-]
			Rowatinex	90 [20]	2.2% (0.6-7.7) [10.0% (2.8-30.1)]	- [-]	- [-]
**Reddit**	492 [75]	6.5% (4.6-9.0) [14.7% (8.4-24.4)]	Allopurinol	49 [8]	12.2% (5.7-24.2) [12.5% (2.2-47.1)]	2.01 (0.79–5.06) [-]	Fisher [-]
			Black seed	17	0.0%	-	-
			Tamsulosin	1134 [232]	21.9% (19.6-24.4) [25.0% (19.9-30.9)]	4.02 (2.74–5.91) *** [1.94 (0.96–3.93)]	Logistic Regression [Logistic Regression]
			Garcinia	38	13.2% (5.8-27.3)	2.18 (0.80–5.96)	Fisher
			Hydrochlorothiazide	46 [6]	8.7% (3.4-20.3) [16.7% (3.0-56.4)]	- [-]	- [-]
			Potassium citrate	509 [147]	13.4% (10.7-16.6) [14.3% (9.5-20.9)]	2.22 (1.43–3.44) *** [0.97 (0.44–2.13)]	Logistic Regression [Logistic Regression]
			Rowatinex	21	14.3% (5.0-34.6)	-	-
